# Myelin Water Fraction Is Transiently Reduced after a Single Mild Traumatic Brain Injury – A Prospective Cohort Study in Collegiate Hockey Players

**DOI:** 10.1371/journal.pone.0150215

**Published:** 2016-02-25

**Authors:** Alexander D. Wright, Michael Jarrett, Irene Vavasour, Elham Shahinfard, Shannon Kolind, Paul van Donkelaar, Jack Taunton, David Li, Alexander Rauscher

**Affiliations:** 1 MD/PhD Program, University of British Columbia, Vancouver, Canada; 2 Southern Medical Program, University of British Columbia Okanagan, Kelowna, Canada; 3 Department of Experimental Medicine, University of British Columbia, Vancouver, Canada; 4 UBC MRI Research Centre, University of British Columbia, Vancouver, Canada; 5 Faculty of Medicine, Division of Neurology, University of British Columbia, Vancouver, Canada; 6 School of Health and Exercise Sciences, University of British Columbia Okanagan, Kelowna, Canada; 7 Faculty of Medicine, Division of Sports Medicine, University of British Columbia, Vancouver, Canada; 8 Faculty of Medicine, Department of Radiology, University of British Columbia, Vancouver, Canada; 9 Department of Pediatrics, Division of Neurology, University of British Columbia, Vancouver, Canada; Instituto Cajal-CSIC, SPAIN

## Abstract

Impact-related mild traumatic brain injuries (mTBI) are a major public health concern, and remain as one of the most poorly understood injuries in the field of neuroscience. Currently, the diagnosis and management of such injuries are based largely on patient-reported symptoms. An improved understanding of the underlying pathophysiology of mTBI is urgently needed in order to develop better diagnostic and management protocols. Specifically, dynamic post-injury changes to the myelin sheath in the human brain have not been examined, despite ‘compromised white matter integrity’ often being described as a consequence of mTBI. In this preliminary cohort study, myelin water imaging was used to prospectively evaluate changes in myelin water fraction, derived from the T2 decay signal, in two varsity hockey teams (45 players) over one season of athletic competition. 11 players sustained a concussion during competition, and were scanned at 72 hours, 2 weeks, and 2 months post-injury. Results demonstrated a reduction in myelin water fraction at 2 weeks post-injury in several brain areas relative to preseason scans, including the splenium of the corpus callosum, right posterior thalamic radiation, left superior corona radiata, left superior longitudinal fasciculus, and left posterior limb of the internal capsule. Myelin water fraction recovered to pre-season values by 2 months post-injury. These results may indicate transient myelin disruption following a single mTBI, with subsequent remyelination of affected neurons. Myelin disruption was not apparent in the athletes who did not experience a concussion, despite exposure to repetitive subconcussive trauma over a season of collegiate hockey. These findings may help to explain many of the metabolic and neurological deficits observed clinically following mTBI.

## Introduction

Impact-related mild traumatic brain injuries (mTBI) are a growing public health concern globally, with major causes including sports, motor vehicle accidents, falls, and assaults. The Centers for Disease Control and Prevention estimate that approximately 1.6–3.8 million mTBI occur annually in the United States alone [1]. Symptoms of concussion, a form of mTBI, are thought to result from mild diffuse axonal injury (DAI) that is not detectable on conventional computed tomography or magnetic resonance imaging (MRI). Currently, the diagnosis and management of this broadly defined and poorly understood injury are based on clinical observation and patient-reported symptoms, despite numerous recent efforts towards the development of objective tools to link functional deficits with quantifiable structural changes. While the majority (80–90%) of individuals with mTBI symptoms recover in 7–10 days, a subset of individuals are left with persistent disability for months to years [2]. Furthermore, emerging evidence has suggested a possible link between clinically manifest concussions, as well as repetitive sub-clinical head impacts, and the development of long-term neurodegenerative changes, termed chronic traumatic encephalopathy [3]. The long-lasting effects of single and repetitive mTBI may include serious cognitive and behavioural deficits, such as problems with affect regulation, attention, memory, and depression [3]. Given these concerns, it is imperative that techniques be developed to provide a more thorough understanding of precisely how mild traumatic brain injuries affect the brain.

Myelin is thought to be an important player in the pathophysiology of TBI, though its role is poorly understood [4,5]; increasingly, evidence implicates that damage to either myelin or the axon can lead to subsequent damage to the other [6,7]. In the case of sport-related head trauma, biomechanical forces imparted on the head cause linear and rotational acceleration-deceleration of the brain within the rigid cranium, which creates diffuse shear strains in the brain tissue. Due to the viscoelastic nature of this tissue, the rate at which such strains are applied is an important factor towards the resultant tissue damage. While primary axotomy is generally observed following high magnitude impacts associated with more severe traumatic brain injuries, it is thought that the axonal pathology observed in mTBI develops over days to weeks following the initial insult [8–10]. Current theories describe a post-traumatic neurometabolic cascade involving ionic flux and indiscriminate glutamate release, leading to mitochondrial dysfunction and calcium sequestration, and a subsequent energy crisis with cytoskeletal damage [9,11]. Intracellular calcium overload has been recognized as a significant cause of damage to both myelin and oligodendrocytes [12]. Impaired axonal transport leads to swellings within the axon containing organelles and other transport materials, creating the potential for a sequence of secondary axonal disconnection and Wallerian degeneration [9,13]. Prolonged global and regional reductions in cerebral blood flow have also been reported following concussion, and were related to recovery duration [14,15]; experimental work has demonstrated that accumulative oxidative stress in the context prolonged cerebral hypoperfusion suppresses both the differentiation of oligodendrocyte precursor cells to oligodendrocytes as well as myelin staining [16]. Thus, loss of oligodendrocytes and corresponding demyelination of affected axons is anticipated [5,7,17]. Histopathologically, this has been observed as axonal bulbs, irregular tortuous axonal varicosities and small globoids of degraded myelin sheath [4,18,19]. While various reports have implicated that myelin fragmentation and degradation occurs following axonal injury [4,18,20–23], dynamic *in vivo* myelin changes have not been directly observed in the human brain. Whether the result of direct, multifocal primary traumatic axonal injury or secondary axotomy, subsequent axonal degeneration is a possible outcome of mild DAI, but axon damage and myelin disruption may be reversible [4,5]. In contrast to situations where strain is focally applied (e.g. at anatomic boundaries), strains distributed more diffusely along the length of an axon create an elongated pattern of axonal swelling, which may be more amenable to intrinsic repair mechanisms [9].

Our current understanding of the pathophysiology of mTBI is derived primarily from post-mortem studies and animal models. While post-mortem studies are useful for exploring histopathological changes in the brain following mTBI, by definition they are unable to provide information on the dynamic post-injury changes explained using animal models. However, it is recognized that the substantial mass and inertia of the human brain play an important role in the development of diffuse axonal injury [9], highlighting an inherent challenge in the development of valid rodent models of mTBI [24]. A non-invasive method for objective, *in-vivo* evaluation of the pathophysiological changes associated with concussion is currently one of the primary goals in mTBI research. Specifically, the dynamic effects of acute mTBI on myelin in the human brain represent a major gap in our understanding of these injuries.

Here, we use myelin water imaging (MWI [25]) to quantify metrics associated with changes in myelin after mTBI. It has previously been identified that decomposing the T2 decay signal results in three components. They include a very long component (~2 seconds) that corresponds to cerebrospinal fluid, an intermediate component (~80 ms) derived from intracellular and extracellular water, as well as a short component (~20 ms) from water that is within the myelin bilayers [26,27]. As such, the fraction of total brain water attributable to myelin can be estimated, and is termed the myelin water fraction (MWF). Recent advances in myelin water imaging have enabled rapid exploration of myelin damage across the whole brain [25]. Previous work has demonstrated a good quantitative relationship between MWF, as derived from MR images, and histological staining for myelin in tissue from both the central nervous system [27] and peripheral nervous system [28]. Being the only technique that has been validated by histopathology, T2-relaxation based myelin water imaging has provided novel insights in demyelinating diseases, such as multiple sclerosis research [29], but has never been used in the context of mTBI to evaluate post-injury myelin dynamics. In this prospective study, we followed a group of individuals at high risk of sustaining a concussion, comprised of two varsity hockey teams, for one athletic season. This is the first study to directly evaluate myelin changes following mTBI in the *in vivo* human brain, assessing myelin water fraction in the context of sport-related head trauma.

Accordingly, our objectives in the current study were to observe changes in myelin water fraction, relative to baseline, at acute, sub-acute, and chronic post-mTBI time points in a group of individuals at high-risk of sustaining a mTBI. We hypothesized that a reduction in myelin water fraction would be observed following concussion, with recovery to near-baseline values by two months post-injury.

## Methods

### Study Design

25 male and 20 female college-aged (mean age 21.2 ± 3.1 years) amateur hockey players from two ice hockey teams participated. All players underwent baseline MRI scanning and neuropsychological testing (Sport Concussion Assessment Tool version 2 (SCAT2, [30]) before the beginning of the hockey season. Players who were diagnosed with a concussion by an independent physician, based on criteria outlined in the 3^rd^ Consensus Statement on Concussion in Sport [30], underwent additional scans and testing at 72-hours, 2-weeks, and 2-months after injury. End of season scans were also completed for the non-concussed cohort. The sample size for this preliminary study was determined from previous cross-sectional literature reports on expected incidence of concussions. Cross-sectional imaging studies have found effects of concussion with less than 15 subjects each [31]. The incidence of concussion in competitive contact sports such as Varsity ice hockey was estimated at 25% or higher [32]. The rationale was then to work with two varsity ice hockey teams where one could expect eight to 12 concussions within a season, sufficient for a preliminary prospective study with pre-injury data from the same brains. Exclusion criteria included a history of severe cognitive impairment, psychiatric or other central neurological disorder, pacemaker use, previous eye surgery, or having worked in an environment likely to expose the participants to the risk of metal fragments being embedded in their eyes. No participants were excluded from this study on these grounds. All participants provided written informed consent prior to participation in the study, which was approved by the University of British Columbia Clinical Research Ethics Board (H11-00423).

### Myelin Water Imaging and Processing

MRI data were acquired on a Philips Achieva 3T scanner equipped with Quasar Dual Gradients and an eight-channel SENSE head coil. A 32 echo T2 scan (14 minutes, 22 seconds) was acquired for myelin assessment (TR = 1000 ms, TE = 10, 20, …, 310, 320 ms, flip angle = 90°, acquisition matrix = 232x192, acquired voxel size 0.99 x0.99 x 5mm, reconstructed voxel size = 0.96 x 0.95 x 2.5mm). The T2 decay was decomposed using a non-negative least squares fit with an extended phase graph algorithm and flip angle optimization [25]. MWF was calculated as T2 signal from 0–40ms divided by the total T2 signal. Other scans acquired during the scanning session included: (a) sagittal 3D T1-weighted scan (TR = 8.1 ms, TE = 3.7 ms, flip angle = 6 degrees, acquisition matrix = 256 x 256 x 160, field of view = 256 x 256 x 160 mm^3^, acquired voxel size = 1 x 1 x 1 mm^3^, reconstructed voxel size = 1 x 1 x 1 mm^3^, SENSE factor of 2 along the left-right direction), (b) sagittal 3D fluid attenuated inversion recovery (FLAIR) (TR = 8000 ms, TI = 2400 ms, TE = 337 ms, flip angle = 6 degrees, acquisition matrix = 256 x 256 x 160, field of view = 256 x 256 x 160 mm^3^, acquired voxel size = 1 x 1 x 1 mm^3^, reconstructed voxel size = 1 x 1 x 1 mm^3^, SENSE factor of 2 along the left-right direction and 2.5 along the anterior-posterior direction), (c) multi echo susceptibility weighted imaging (SWI) using an axial 3D gradient echo scan (TR = 36 ms, TE = 6/12/18/24/30 ms, flip angle = 17 degrees, acquisition matrix = 440 x 222 x 64, field of view = 220 x 166 x 128 mm^3^, acquired voxel size = 0.5 x 0.5 x 2 mm^3^, reconstructed voxel size = 0.5 x 0.5 x 1 mm^3^, SENSE factor of 1.2 along the left-right direction), and (d) diffusion tensor imaging (DTI) scan (TR = 7015 ms, TE = 60 ms, flip angle = 90 degrees, acquisition matrix = 100 x 99, field of view = 224 x 224 x 154 mm^3^, acquired voxel size = 2.2 x 2.2 x 2.2 mm^3^, reconstructed voxel size = 2 x 2 x 2.2 mm^3^, SENSE factor of 2.1 along the anterior-posterior direction, b_0_ = 0, b_1_ = 700 sec/mm^2^, 60 noncolinear directions). Total data acquisition time was 48 minutes. Results from the conventional scans are reported elsewhere [33].

### Statistical Analysis

Myelin water fraction changes were evaluated through comparison of concussed athletes’ baseline scans to those acquired at 72 hours, 2 weeks, and 2 months post-injury. Impact-related mTBI is a very heterogeneous injury [34]. However, in the current preliminary study, the intent was to prospectively investigate myelin dynamics after mTBI without making any clinical predictions on an individual basis, let alone correlate clinical measures with imaging measures. Voxelwise statistical analysis of the data was performed using tract-based spatial statistics (TBSS) [35], part of the Functional MRI of the Brain Software Library (FSL [36]), which creates a white matter skeleton for each participant’s brain using fractional anisotropy (FA) maps obtained from diffusion tensor images. First, FA images were created by fitting a tensor model to the raw diffusion data using FDT, and then brain-extracted using BET. All subjects' FA data were then aligned into a common space using the nonlinear registration tool FNIRT, which uses a b-spline representation of the registration warp field. Next, the mean FA image was created and thinned to create a mean white matter skeleton that represents the centres of all tracts common to the group. MWF maps were then registered into DTI space and projected onto the white matter skeleton. Voxelwise statistics were carried out using the randomise tool in FSL for 5000 permutations with threshold-free cluster enhancement to assess differences in myelin water fraction at each post-injury time point compared to baseline, while controlling for, as explanatory variables, age, gender, SCAT2 results, and whether scans were acquired before or after an upgrade to the scanner's gradient system occurred. The locations of significant voxels were identified anatomically using the John’s Hopkins University standard white matter atlas. No outliers were excluded from analysis, as possible “outliers” may well be within the normal spectrum of mTBI. Corrected level of significance was set at alpha = 0.05. These methods were chosen explicitly to examine diffuse changes in brain structure, without *a priori* assumptions as to where such changes would occur, while compensating for multiple comparisons. This approach effectively restricts analysis to areas that are injured in the majority of subjects, and follows the myelin signal in these brain areas. Analysis of the data was performed by non-supervised algorithms. The study had a conventional imaging component as well, which required the reading of MRI data by radiologists. These data will be reported elsewhere.

## Results

**Fig 1:**
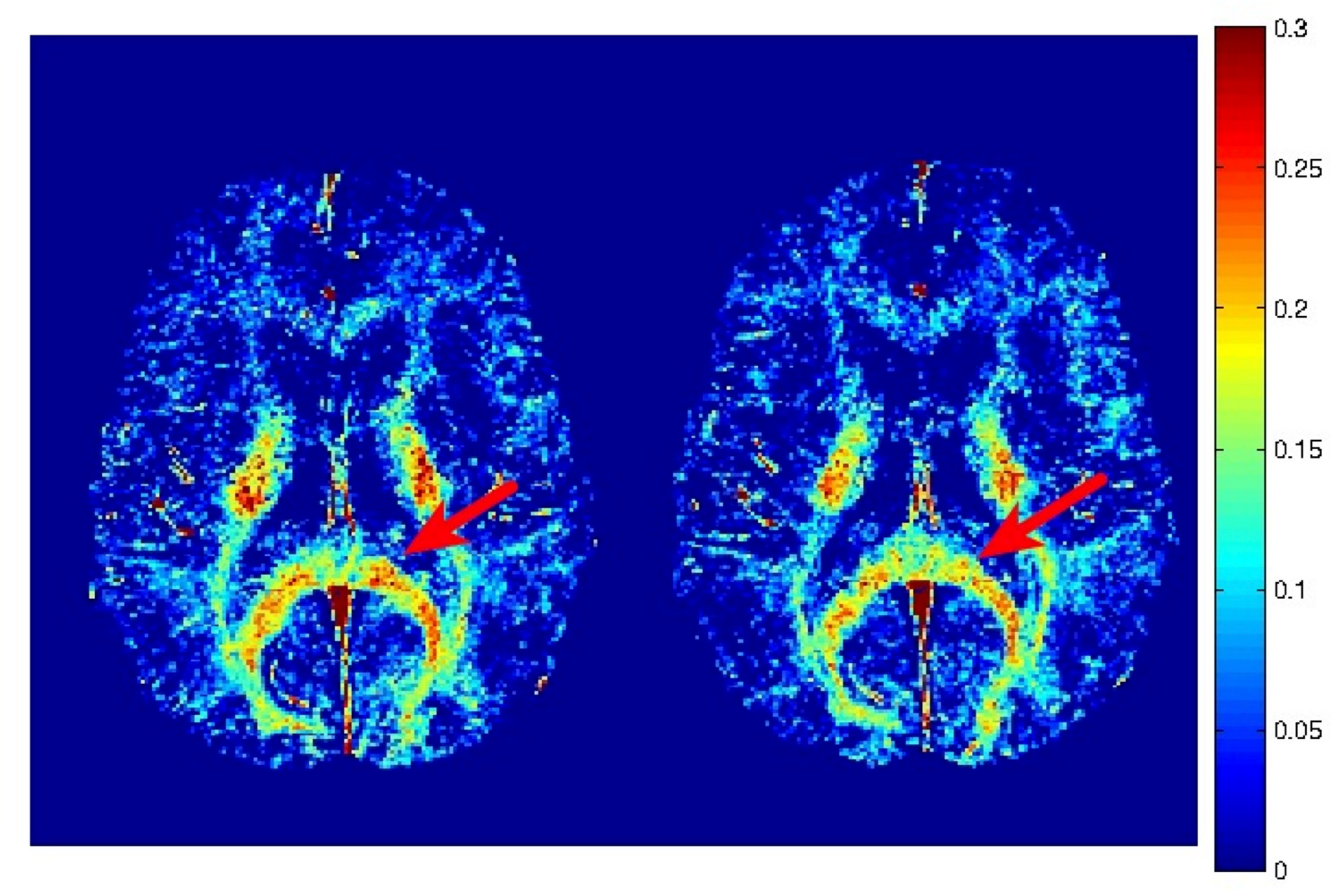
Representative myelin water fraction maps. Myelin water fraction maps from a concussed athlete at baseline (left) and two weeks post-injury (right). Myelin water fraction is measured as the T2 signal from 0–40 ms divided by the total T2 signal. A region of the corpus callosum with a visible reduction in MWF post-injury is highlighted by the red arrow.

**Fig 2:**
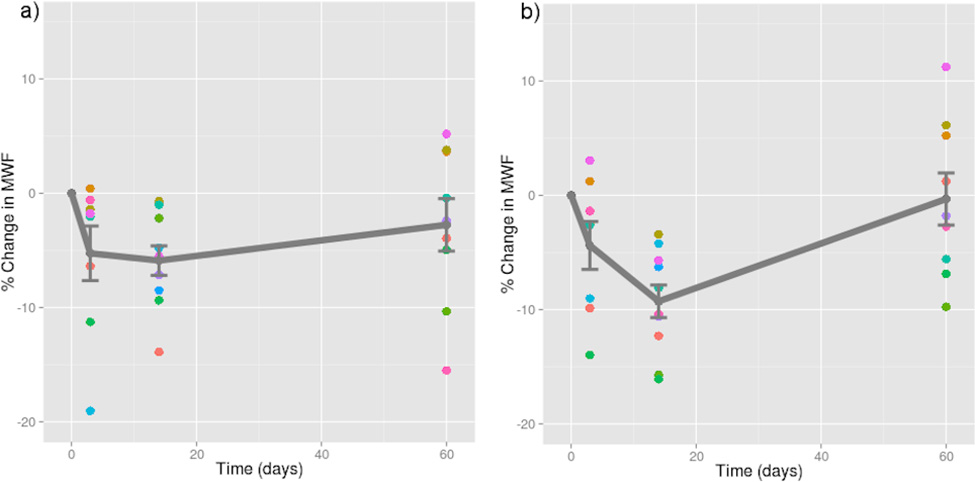
Relative myelin water fraction change post-injury. Change scores for myelin water fraction, relative to baseline, plotted against time for each subject with a mild traumatic brain injury in all significant voxels A) across the whole brain; B) in the splenium of the corpus callosum (a structure most commonly affected in mild TBI). Dots represent data points for each injured athlete (mean ± standard error plotted in grey). Note: time zero refers to baseline.

**Fig 3:**
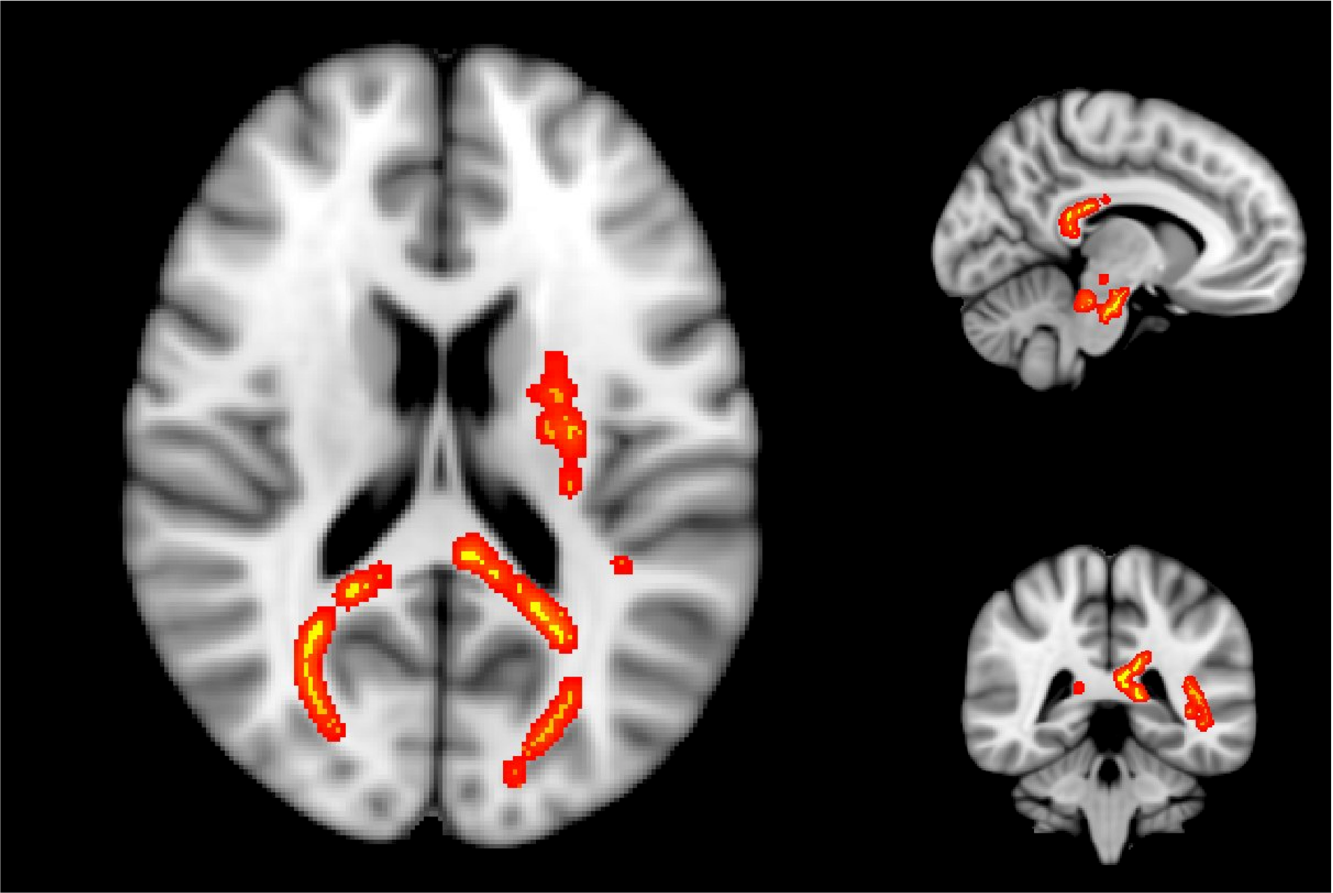
Brain areas with significantly reduced myelin water fraction. Areas of significantly reduced myelin water fraction in athletes with concussion at two weeks post-injury, superimposed on a standard brain. These areas include the splenium of the corpus callosum, right posterior thalamic radiation, left superior corona radiata, left superior longitudinal fasciculus, and left posterior limb of the internal capsule.

11 players (six female) sustained a concussion during the ice hockey season (Table 1). When comparing baseline scans between concussed and non-concussed athletes, voxelwise TBSS revealed no significant differences in myelin water fraction. Within the non-concussed cohort, no significant differences were identified between pre-season and post-seasons scans. Eight out of 11 concussed subjects were able to complete scanning at 72 hours post-injury, 10 out of 11 athletes were scanned at 2 weeks, and nine out of 11 were scanned at 2 months post-injury (Fig 1; raw MWF values for concussed subjects at each scan are contained within S1 Table). Within the concussed cohort, TBSS showed clusters of voxels with significantly reduced myelin water fraction at two weeks post-injury, relative to baseline (Fig 2). These voxel clusters were located in the splenium of the corpus callosum, right posterior thalamic radiation, left superior corona radiata, left superior longitudinal fasciculus, and left posterior limb of the internal capsule (as shown in Fig 3). Across all significant voxels, this represented a 5.9% ± 1.2% (mean ± standard error) reduction in MWF from baseline to 2 weeks. A decrease in MWF was observed at 72 hours post-injury in these voxels, but this did not achieve statistical significance (p = 0.076). No significant MWF changes were observed between baseline and 2 months post-injury.

**Table 1:** Demographic Characteristics for All Athletes.

Subject	Concussed (Y/N)	Sex	Age
1	Y	M	22
2	Y	M	21
3	Y	F	21
4	Y	F	19
5	Y	F	22
6	Y	F	21
7	Y	M	22
8	Y	M	24
9	Y	F	19
10	Y	F	19
11	Y	M	23
12	N	F	23
13	N	M	21
14	N	F	18
15	N	F	20
16	N	F	18
17	N	F	18
18	N	M	21
19	N	M	24
20	N	F	21
21	N	M	22
22	N	M	25
23	N	M	22
24	N	M	21
25	N	F	19
26	N	F	18
27	N	M	22
28	N	M	20
29	N	M	21
30	N	M	21
31	N	F	20
32	N	M	22
33	N	F	18
34	N	M	23
35	N	F	17
36	N	M	20
37	N	F	19
38	N	F	17
39	N	M	21
40	N	M	23
41	N	F	36
42	N	M	18
43	N	M	22
44	N	M	23
45	N	M	26

## Discussion

In the current preliminary report, we present the first prospective study on myelin water fraction in the context of mTBI in the human brain, and demonstrate significant post-injury reductions. In contrast, tract-based spatial statistics of post-season MWF maps demonstrated no differences from pre-season scans in athletes who did not sustain a concussion. Myelin water fraction, derived from the T2 decay signal, is a direct marker of myelin content in the brain. These data may indicate a process of transient myelin disruption following mTBI in areas of the brain previously shown to exhibit diffusivity changes (reviewed in [37]). Furthermore, MWF recovered to near-baseline values by 2 months post-injury. Although significantly reduced MWF values were observed at the 2-week time point only, there was a trend towards decreased MWF at 72 hours post-injury (p = 0.076). That no differences were seen between pre- and post-season for non-concussed athletes is encouraging, given recent concern over the effect of exposure to repetitive sub-clinical trauma [38].

The exact time course of myelin alterations following sport-related concussion cannot be determined from this exploratory study. As the subjects were acutely concussed and enduring the worst phase of their symptoms, only eight of the 11 subjects were able to complete the 72 hour scan; it is possible that the reduced sample size at this time point may have obviated statistical significance from being achieved. Previous work has demonstrated that peak concentrations of myelin basic protein in the cerebrospinal fluid (CSF) occur at 48–72 hours following severe TBI, where primary axotomy likely occurred [39]. In milder forms of TBI such as concussion, where primary axotomy does not occur, myelin degradation (as a downstream effect of the post-injury neurometabolic cascade and subsequent neuroinflammation) may take longer to occur, which may also explain the why the MWF reduction at 72 hours post-injury did not achieve statistical significance. It is important to point out that, although we observed a significant reduction in MWF at 2 weeks post-injury, peak reductions in myelin content may have occurred earlier or later. Indeed, significant loss of myelin for up to 21 days following injury has been reported following fluid percussion injury in rats [17], although this injury model is recognized to be of greater severity than sport-related concussion. Maxwell and colleagues demonstrated that optic nerve fibre degeneration may be initiated days to weeks after a mechanical stretch injury [10]. Using closed-skull impacts in mice (an injury model more similar to concussion), Mierzwa et al. [4] reported markers of neuroinflammation localized to areas of axon and myelin pathology for up to 6 weeks. Similarly, true recovery of MWF to baseline values may have occurred earlier or later than two months following injury. Myelin debris is known to stimulate neuroinflammatory processes [4,23]. In the context of Wallerian degeneration, myelin debris has been reported to remain for several months before full clearance by macrophages [40], while previous work has shown myelin fragments contained within activated microglia for up to 4 weeks after fluid percussion injury [41]. Remyelination of multiple sclerosis lesions has also been observed within 2 months [42]. The exact time course of myelination dynamics following mTBI in humans is not currently known. Nevertheless, the authors of the current preliminary article postulate that myelin fragmentation and degeneration led to the observed reductions in MWF, with the recovery of MWF by two months post-injury likely due to remyelination of the affected axons by oligodendrocytes.

We are inferring that changes in myelin water fraction are indicative of true changes in myelination within the brain. T2 relaxation-based MWI provides insight into tissue characteristics that are not observed using standard MR imaging techniques. Numerous studies have supported that the short component of the multicomponent T2 relaxation distribution is specific to myelin, and have suggested this as the best imaging technique to distinguish between myelination and inflammation [43–46]. Post-mortem MRI-pathology correlation studies in both CNS [27] and animal peripheral nerve [28] have demonstrated good quantitative and qualitative relationships between MR-derived MWF and histological staining for myelin. Myelin water imaging was shown to have high reproducibility in healthy brains, both longitudinally and between imaging sites using the same scanner type and the same protocol [47,48]. Moreover, MWF in the normal appearing white matter of people with multiple sclerosis was also shown to be stable over at least six months [49]. In a guinea pig model of demyelination (experimental allergic encephalomyelitis), a decrease in the short T2 component was observed, which was consistent with histological myelin loss [43]. As such, it is reasonable to conclude that the MWF changes observed in the current study are indeed reflective of transient reductions in myelin content following mTBI.

The current results provide the first direct, *in-vivo* demonstration of myelin damage following sport-related mTBI in humans, a notion that is supported by previous work in different models. Experimental models have implicated myelin disruption and subsequent remyelination to be major components of the degeneration and recovery process in white matter following mTBI [4,5]. Multiple mTBI studies using closed-skull impacts in mice have shown significant demyelination distributed across intact axons using electron microscropy and histological staining techniques. Donovan and colleagues observed structural abnormalities in rats that experienced controlled cortical impacts, including separation of myelin from the axon, as well as decompaction and fragmentation of the myelin sheath following repetitive mTBI, compared to sham injury [50]. Both single and repetitive injuries have been shown to lead to significant reductions in the thickness of myelin surrounding axons [4,50]. Observations of increased Luxol fast blue staining (a histological stain for myelin) within the cytoplasmic compartment of cells following traumatic brain injury has supported the role of myelin degeneration and subsequent active phagocytosis of myelin fragments [20]. Reports of acute decreases in axial diffusivity following experimental closed-skull mTBI, measured using diffusion tensor imaging, followed by delayed increases in radial diffusivity also imply that axonal damage may be followed by myelin damage [50,51]. Myelin disruption slows signal conduction, thereby desynchronizing circuitry within the brain [52] that may contribute to the observed neurological deficits that accompany mTBI, such as reduced information-processing speed. Accordingly, demyelination has been proposed to explain significantly slower interhemispheric transfer times (a measure of conduction speed through the corpus callosum) in paediatric human TBI patients, who also exhibited lower fractional anisotropy and higher mean diffusivity values in at least 13 out of 18 white matter tracts that were evaluated [53].

Transient reductions in the myelination status of axons following mTBI, as observed in the current study, may also help to explain the observation of a ‘temporal window of vulnerability’ following concussion [54], by which the brain is more sensitive to additional trauma during the recovery period. Magnetic resonance spectroscopy findings have demonstrated significant reductions in N-acetyl-aspartate (NAA) in the brain [54,55], a marker recently shown to be localized in the myelin of adult brains [56]. Such NAA changes largely resolved within 30 days, but they did not coincide with clinical resolution of symptoms, implying a period of acute metabolic imbalance during the post-mTBI recovery process [54,55]. Interestingly, MR spectroscopy studies in multiple sclerosis patients have also detected significant NAA reductions in the absence of any visible lesions [57]. The integrity of axonal neurofilaments is dynamically regulated by the influence of myelin on kinase and phosphatase activity. From a developmental perspective, myelin increases phosphorylation of neurofilaments, enabling lateral extension of these structural proteins, which correspondingly increases axonal calibre and conduction velocity [58]. Animal studies have shown that demyelination leads to a reduction in neurofilament phosphorylation and consequent axon calibre [59,60]. While the role of the myelin sheath in facilitating propagation of electrical impulses is well known, its pivotal role in neuronal metabolism is highlighted by estimates that myelinated axons are up to 70 times more metabolically efficient than unmyelinated axons [61]. Myelin may also modulate the activity of the enzyme that hydrolyzes NAA into aspartate and acetate (involved in the production of coenzyme A) [62]. As such, post-mTBI metabolic disturbances may at least partly be explained by changes in myelination [7]. Moreover, previous research has suggested that unmyelinated axons may be more vulnerable to trauma, demonstrating greater electrophysiological impairment than their myelinated counterparts [63,64]. It is possible that myelination changes after concussion may sensitize axons to further damage if exposed to additional trauma [21].

In evaluating changes in myelination within the brain, neuroimaging techniques that enable us to reliably observe pathophysiological changes behind an intact blood brain barrier, such as myelin water imaging, are more appropriate than measurement of blood and CSF biomarkers. An important clinical limitation of using CSF for biomarker analysis is the invasive nature of acquiring such samples, which requires a lumbar puncture. While acquiring blood samples is less invasive, the blood-brain barrier, which is thought to remain intact following mTBI [65,66], attenuates the ability of biomarkers to enter the blood. Some biomarkers may be bound to carrier proteins or undergo degradation in the blood, further decreasing their measurable concentration, while extracerebral sources of some biomarkers also exist. Consequently, it has been difficult to identify reliable blood markers for mTBI [67]. With specific regards to the evaluation of myelin, myelin basic protein (MBP) is the best available fluid biomarker of myelin integrity. No studies are currently available regarding the effects of mTBI on MBP levels in the CSF. While the specificity of MBP in the blood for TBI appears to be adequate (96%), this marker lacks the sensitivity (44%) required to be clinically useful [68]. In contrast, myelin water imaging enables us to reliably observe pathophysiological reductions in myelin behind an intact blood brain barrier, and thus appears to be a more appropriate *in-vivo* method towards answering research questions related to changes in myelination.

Myelin water imaging is a preferable technique to diffusion tensor imaging (DTI) for the evaluation of myelin integrity following mTBI [46]. DTI is sensitive to diffusion properties of the various tissues within the brain, and has often been used as an indirect marker of myelin integrity in mTBI research. In white matter, the neurofibrils, axonal membrane, and myelin sheath promote water diffusion axially (along the length of the axons) relative to perpendicular flow, a phenomenon termed anisotropic diffusion [69] and quantified as fractional anisotropy (FA). Reports on diffusivity changes following mTBI have been equivocal, and have been reviewed in detail previously [37,70,71]. While many studies have reported a decrease in FA following mTBI [72–74], numerous other studies have demonstrated an increase in FA [31,75,76], while other studies have reported no changes [77,78], or FA changes in both directions [76,79]. At least part of these discrepancies may be related to inconsistent injury-to-scan intervals across studies, as the time course of changes in FA post-injury is not fully understood. Nevertheless, many studies infer that changes in DTI metrics reflect compromises in white matter and, more specifically, myelin integrity [53]. Such interpretations must be made with caution, however, as myelin changes are not necessary or sufficient to yield changes in metrics of anisotropic diffusion [69]. In fact, a fundamental limitation of interpreting changes in DTI metrics is the inability of this technique to distinguish between multiple factors that influence them, including fiber diameter and density, fiber orientation, membrane permeability, edema, and demyelination [80]. In contrast, T2 relaxation-based myelin water imaging *directly* evaluates myelin content in the brain, and has been validated histopathologically [27,46,81].

There are several limitations to the current preliminary study. Firstly, the sample size of concussed athletes is relatively small, which precludes an in-depth analysis of any differences between male and female athletes, or the relationship between myelin water fraction and neuropsychological scores. The prospective nature of this preliminary study limited the sample size but allowed us to directly compare intra-individual changes before and after injury. Nevertheless, the authors feel that this approach is preferable to a cross-sectional case-control design with a higher sample size. By definition, the specificity of the myelin water imaging technique to myelin comes at the expense of a decreased signal-to-noise ratio, as myelin water accounts for only ~15% of total brain water content (Sled et al. 2004 in [46]). Further, different individuals have different degrees of myelination within their white matter. As such, it is imperative to obtain pre-injury data when using this imaging approach to mitigate the effects of inter-individual variation. Unfortunately, not all subjects completed all MRI scans. However, the statistical power is similar at all time points with eight out of 11 concussed subjects scanned at 72 hours, 10 out of 11 scanned at two weeks, and nine out of 11 scanned at two months. An upgrade to the MRI scanner gradient system occurred during the course of the study, which included a replacement of the gradient coil. The exact date of this upgrade was known and was included as a covariate in the statistical analysis, but did not alter the results. Furthermore, monthly scans of a geometry phantom were acquired during this study, which were evaluated for field distortions due to gradient non-linearities; no such distortions were found in the phantom data. Our statistical approach (TBSS) is exploratory in nature, unable to elucidate subject-specific MWF changes as it limits analysis to the central cores of large WM tracts common across all subjects. Therefore, TBSS also does not examine the grey matter-white matter junction, where traumatic axonal injury also occurs. Nevertheless, the fact that significant MWF changes are seen in a preliminary study with a small sample size emphasizes the need for more in-depth prospective investigations into the effects of mTBI on myelin dynamics, including analytical approaches to assess within-subject changes [34,79].

## Conclusions

In order to mitigate both the incidence and severity of concussive-type mild traumatic brain injuries, a better understanding of their neurobiological underpinnings is needed. This will facilitate the development of objective tools to improve the detection and management of these injuries. Although not currently appropriate for clinical application, myelin water imaging appears to be a promising approach to understanding the role of myelin following sport-related concussion. In this exploratory study, we detected significant reductions in myelin content in the brain of concussed athletes 2 weeks following injury, which recovered by 2 months. While validation is required in a larger number of subjects, the results of this preliminary study may indicate a process of transient myelin disruption following mTBI, and encourage the development of additional investigation into the effects of repetitive concussive and subclinical head impacts on the time course of myelination changes in the brain. Furthermore, exploration of the relationships between age, sex, and myelination on susceptibility to injury, as well as between myelin status and neurocognitive performance, is warranted.

## Supporting Information

S1 Table Raw myelin water fraction values across concussed athletes for each scan. Each column represents raw myelin water fraction data from a given subject (S##) at baseline (BL), 72 hours (72H), 2-weeks (2W), or 2 months (2M) post-injury.
(ZIP)
